# Classification and Prediction by Pigment Content in Lettuce (*Lactuca sativa* L.) Varieties Using Machine Learning and ATR-FTIR Spectroscopy

**DOI:** 10.3390/plants11243413

**Published:** 2022-12-07

**Authors:** Renan Falcioni, Thaise Moriwaki, Mariana Sversut Gibin, Alessandra Vollmann, Mariana Carmona Pattaro, Marina Ellen Giacomelli, Francielle Sato, Marcos Rafael Nanni, Werner Camargos Antunes

**Affiliations:** 1 Plant Ecophysiology Laboratory, Graduate Program in Agronomy, Department of Agronomy, State University of Maringá, Av. Colombo, 5790, Maringá 87020-900, Brazil; 2 Optical Spectroscopy and Thermophysical Properties Research Group, Graduate Program in Physics, Department of Physics, State University of Maringá, Av. Colombo, 5790, Maringá 87020-900, Brazil

**Keywords:** anthocyanins, carotenoids and chlorophylls, discriminant analysis, flavonoids, phenolic compounds, pigment analysis, PLSR analysis, phytochemistry, secondary metabolism

## Abstract

Green or purple lettuce varieties produce many secondary metabolites, such as chlorophylls, carotenoids, anthocyanins, flavonoids, and phenolic compounds, which is an emergent search in the field of biomolecule research. The main objective of this study was to use multivariate and machine learning algorithms on Attenuated Total Reflectance Fourier Transform Infrared Spectroscopy (ATR-FTIR)-based spectra to classify, predict, and categorize chemometric attributes. The cluster heatmap showed the highest efficiency in grouping similar lettuce varieties based on pigment profiles. The relationship among pigments was more significant than the absolute contents. Other results allow classification based on ATR-FTIR fingerprints of inflections associated with structural and chemical components present in lettuce, obtaining high accuracy and precision (>97%) by using principal component analysis and discriminant analysis (PCA-LDA)-associated linear LDA and SVM machine learning algorithms. In addition, PLSR models were capable of predicting Chl*a*, Chl*b*, Chl*a*+*b*, Car, AnC, Flv, and Phe contents, with R^2^_P_ and RPD_P_ values considered very good (0.81–0.88) for Car, Anc, and Flv and excellent (0.91–0.93) for Phe. According to the RPD_P_ metric, the models were considered excellent (>2.10) for all variables estimated. Thus, this research shows the potential of machine learning solutions for ATR-FTIR spectroscopy analysis to classify, estimate, and characterize the biomolecules associated with secondary metabolites in lettuce.

## 1. Introduction

Plants produce many secondary metabolites as a function of species, genotype, and environmental and physiological conditions [1,2,3,4]. These secondary compounds may be responsible for important functions in plant biology [5,6,7]. The classification and prediction of secondary metabolite contents that are rich in biological activities is of great interest to researchers [2,3,8]. Thus, to elucidate the variation in many secondary compounds or metabolites based on secondary metabolism, high-throughput and large-scale data analyses have been used in recent years [9,10,11,12]. In this sense, one of the main methods of characterization and analysis with satisfactory precision and accuracy has been used by the combination of different FTIR-spectroscopic techniques and machine learning tools [11,13,14,15,16,17].

Many molecules and biocompounds, such as secondary metabolites, including flavonoids, alkaloids, phenylpropanoids, terpenes, carotenoids, amino acids, and other phenolic compounds, have been well explored because some of these compounds possess alimentary or pharmaceutical properties that are attractive to human health or affect plant metabolism [2,18,19]. Flavonoids/phenolics represent one of the most variated groups of secondary metabolites in the plant kingdom [3,20,21] and include 6000 known chemical structures [18,22,23]. Phenolic compounds, flavonoids, anthocyanins, carotenoids, and chlorophylls have high economic and production values [2,7,24,25,26]. The structural diversity of these compounds contributes to their numerous physiological activities on plants, such as their advantage to plant adaptation to distinct environmental conditions, such as cold or heat, many biotic interactions and pathogen attacks, and light (high intensity) and UV stresses [18,23,27,28]. In addition, their unique vibrational bands can be spectroscopically detected. In this sense, there is much interest in the analysis of leaves, fruits, seeds, stems, tubers, roots, and flowers [18,29,30]. Furthermore, green, green-purplish, or full purple lettuce varieties represent one of the most economically important and popular vegetables consumed, for which global production is estimated to be 27 million tons worldwide (FAO, 2022).

ATR-FTIR equipment is a sensor based on spectroscopy measurements that can be used to characterize the properties of molecules. It can be utilized for crops and food [3,19,29,30,31,32]. The prediction of biomolecules from spectroscopy tools is based on the vibrational relationships of infrared with molecular bonds and, mainly, its vibrational modes, as well as C=O stretching, –C–H bonds, aromatic C=C, –COOH, –NH_3_, –C–H, and –O–H. The output is a spectrum of functional group vibrations at specific wavenumbers [3,4,7,22]. Thus, based on horticultural crops and postharvest, chemometric parameter analysis of many vibrational groups combined with machine learning algorithms makes it possible to define better strategies for vertical farms, industries, and consumers. For example, consider the digital revolution 4.0, in which an economy of US $750 billion a year is estimated. Thus, machine learning in decision making and analysis by ATR-FTIR and other spectroscopy tools could be a promising approach by which to classify and predict biomolecules. In this sense, it could mitigate problems with prediction and training and avoid economic losses [31,32].

**Figure 1:**
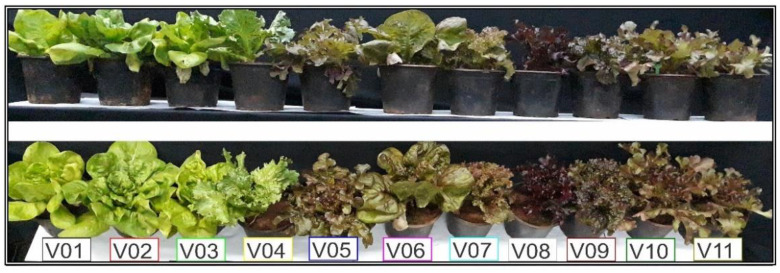
Images of lettuce (*Lactuca sativa* L.) plants evaluated in this study, displaying significant bioaccumulation of pigment variations (accumulation of the pigments based secondary metabolism). Upper panel, details of the lateral profile. Lower, detail of the plant leaves. The box displays eleven different genotypes of lettuce: V01—Rainha de Maio, V02—Vitória, V03—Maravilha de Inverno, V04—Grandes Lagos Americana, V05—Mimosa Prado, V06—Quatro Estações, V07—Batávia Joaquina, V08—Mimosa Vermelha, V09—Batávia Cacimba, V10—Pipa and V11—Mimosa Rubi.

Many studies have explored multivariate statistical methods to directly model frequently used plant chemometrics as a function of machine learning algorithms [11,13,33,34,35]. Developments in the portability, accuracy, and sensitivity of ATR-FTIR coupled computational algorithms and multivariate tools for modelling and other statistical-machine learning methods [3,4,28] have allowed advances in classifying and predicting the variety of plants. Thus, this approach is used to evaluate a variety of commonly investigated plant secondary metabolite chemometrics processes based on vibrational groups and specific band properties [26,34,36,37,38,39]. In this sense, a calibration model is developed by combining leaf spectra collected on a uniform and calibrated spectral source in a consistent manner with independent and reference methods for measuring samples. Subsequently, model-based ATR-FTIR and machine learning algorithms were developed and validated by comparing relationships between observed and predicted values collected from other independent databases, samples, or environments of field experiments [4,7,11,36]. Furthermore, an adjustment model was then used to predict the variable of interest in uncharted samples based on their many spectral spectroscopy signatures. Currently, ATR-FTIR spectroscopy analysis uses the full or ranged (specific) spectrum for the analysis of many chemometric varieties in plants [7,22,33,36,40] to quantify a particular bond. From this perspective, infrared spectroscopy has been highly successful in both inorganic and organic chemistry, and one application is shown here to monitor biomolecules presenting different and principal pathways of secondary metabolism in lettuce (Figure 1).

**Figure 2:**
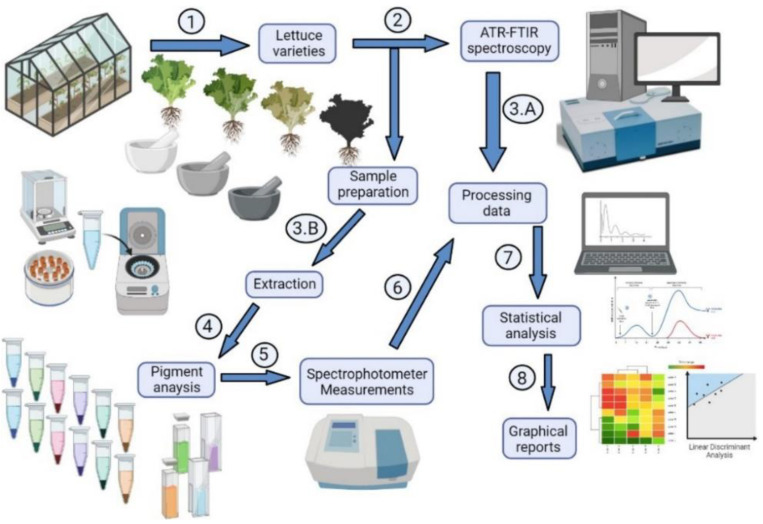
Flowchart describing classified and predicted pigments in *Lactuca sativa* L. (lettuce varieties) using machine learning and ATR-FTIR spectroscopy for analysis. (1) Greenhouse to grow the lettuce; (2) sample preparation and analysis; (3. A) ATR-FTIR spectroscopy direct analysis; (3. B) material for chemical quantification; (4) pigment extractions in different solvents; (5) analysis in a microreader or spectrophotometer; (6) data processing and multivariate statistical analysis based on machine learning; (7) modelling, calibration, classification, and prediction models; and (8) training, testing, and graphical reports by analysis.

Considering the needs described above and the importance of lettuce plants, the main objective was to assess the capacity to classify and predict seven functional molecules, such as chlorophylls, carotenoids, anthocyanins, flavonoids, and phenolic compounds, in eleven lettuce varieties based on a machine learning algorithm (PCA, LDA, and SVM) and ATR-FTIR spectroscopy (4000-400 cm^−1^) to characterize and distinguish plant secondary metabolites. A full spectrum of lettuce variety plants was obtained (4000-400 cm^−1^). In some cases, the range (1500-1150 cm^−1^) was used as a rapid dataset to estimate seven principal chemometric attributes to classify and predict chlorophyll *a* (Chl*a*), chlorophyll *b* (Chl*b*), total chlorophylls (Chl*a*+*b*), carotenoids (Car; total carotenes and xanthophylls), anthocyanins (AnC), flavonoids (Flv), and phenolic compounds (Phe). If successful, this approach could be directly used to classify important biomolecule-based chemometric and fingerprint parameters in lettuce varieties with higher accuracy and precision to predict model-based machine learning (Figure 1 and Figure 2).

## 2. Results

### 2.1. Descriptive Analysis

The descriptive analysis of the seven classes of compounds (chlorophylls (*a*, *b*, and *a* + *b*), total carotenoids, anthocyanins, flavonoids, and phenolics) in different lettuce varieties is displayed in Table 1. The CVs (%) ranged from 16.79 to 126.53% (Table 1); 01 of the 07 parameters analyzed demonstrated CVs (%) labelled as medium to very high (Phe) and six very high parameters (Chl*a*, Chl*b*, Chl*a*+*b*, Car, AnC, and Flv) (see Abbreviation list), allowing, as broad as possible, to encompass natural variability, mainly for AnC contents.

### 2.2. Cluster Heatmap Analysis

**Figure 3:**
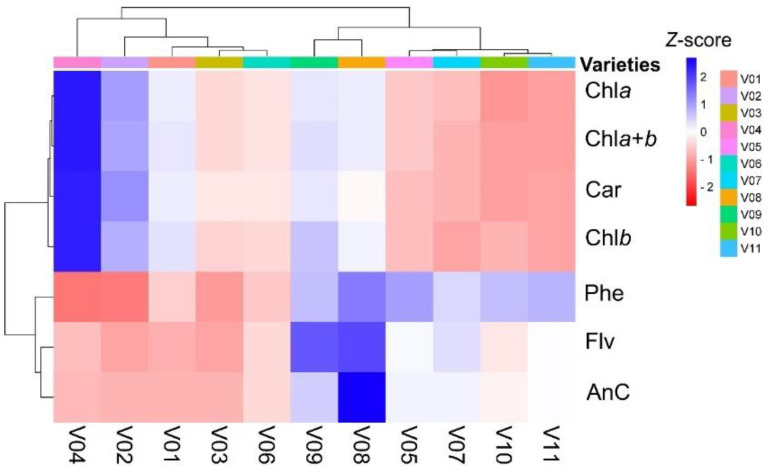
Cluster heatmap summarizing pigment contents from eleven varieties of *Lactuca sativa* L. (lettuces). Numerical differences within the data matrix are shown by the Z score of the color by scale red to blue indicate decreased to increased values, respectively. Parameters are clustered in the rows; sample groups are clustered in the columns by pigment content in leaves at independent factors. See varieties in Figure 1 or Abbreviation list. (*n* = 132 samples).

The cluster heatmap grouping lettuce varieties based on the chemometric parameters evaluated is shown in Figure 3. The heatmap separates the green, green-purplish, and purple varieties of lettuce, evidencing a rise from the green pigments (V01, V02, V03, and V04), intermediate contents (V05, V07, V10, and V11), and the highest purple pigments (V08 and V09), distinguishing the highest chlorophyll and anthocyanin accumulation (Figure 3), except for V06, which is naturally “centered” among distinct chemometrics data (Figure 1 and Figure 3). The lowest contents of Phe, Flv, and AnC were observed in green lettuce, and the highest were observed in purple varieties. On the other hand, clusters formed with varieties that accumulated the lowest levels of Flv, AnC, and Phe (blue squares) were related to greater accumulation of Chl*a*, Chl*b*, Chl*a*+*b*, and Car (red squares) (Figure 3).

### 2.3. ATR-FTIR Spectroscopy Analysis

**Figure 4:**
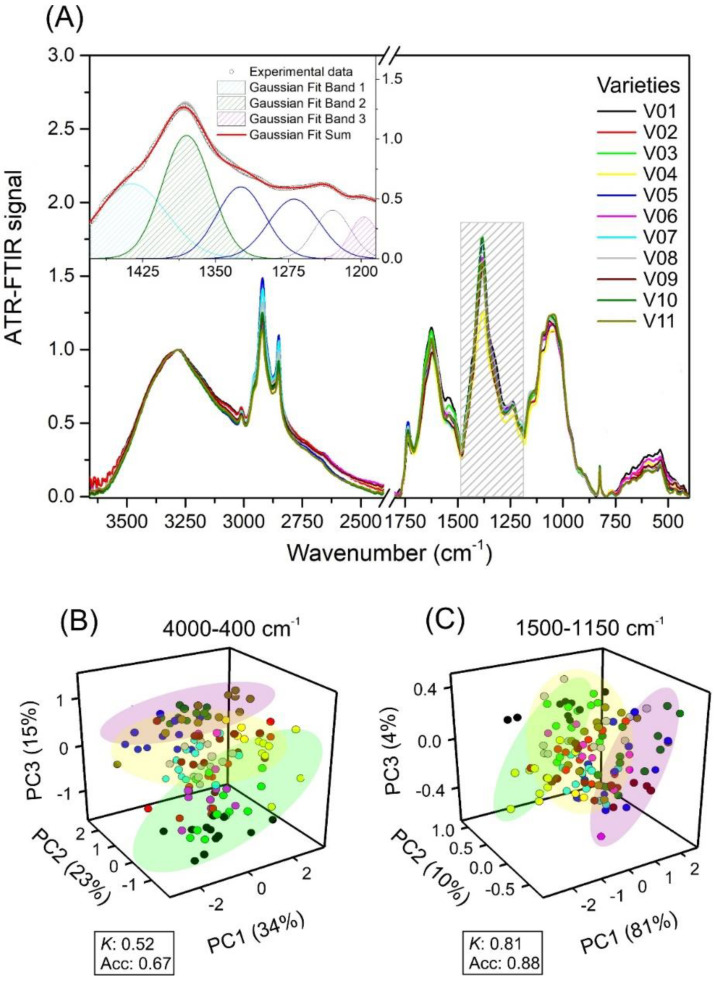
(**A**) Average ATR-FTIR signal profile of eleven varieties of lettuce over the full range (4000-400 cm^−1^) of ATR-FTIR spectroscopy and ranged from 1500 to 1150 cm^−1^. The inset displays the experimental data and Gaussian fit curves (1436, 1380, and 1197 cm^−1^). (**B**) 3D plot of the principal component analysis score for the first three principal components (PC1, PC2, and PC3) of the spectroscopy data (4000-400 cm^−1^). (**C**) 3D plot of the principal component analysis score for the first three principal components (PC1, PC2, and PC3) of the spectroscopy data (1500-1150 cm^−1^). Data collected from lettuce plants. Green, yellow, and purple colors display correlation clusters of lettuce varieties that showed similar groups of pigments in leaves. *K*: *Kappa* and Acc: accuracy. The ATR-FTIR signal intensity was normalized at 3270 cm^−1^ (OH stretching band).

ATR-FTIR spectroscopy data for the eleven lettuce varieties (132 samples) are displayed in Figure 4A. PERMANOVA was shown to discriminate significant wavenumbers (F: 6.73; *p* < 0.001) from the spectra. Most functional groups (vibrational groups) detected in the full range (4000 to 400 cm^−1^) showed differences between varieties in ATR-FTIR signals. A slight and significant variation in ATR-FTIR signal factor intensity was observed in a Gaussian fit based on leaf pigments, such as carotenoid and chlorophyll, and structural differences in the organic molecules of leaf mesophyll (Figure 4A).

The selected spectral range (1500 to 1150 cm^−1^) is highlighted in the inset of Figure 4A. Gaussian and functional adjustment were accomplished to explore the main differences in vibrational modes of the secondary metabolites in these regions. Three main bands (1436, 1380, and 1197 cm^−1^; inset) showed significant differences. For example, there was a minor correlation at 1436 and 1380 cm^−1^ for flavonoids, anthocyanins, or phenolic compounds, and a higher correlation (r = 0.960; *p* < 0.001) at 1197 cm^−1^ with anthocyanins (Figure 4A; inset). The variability explained based on the first three PCA scores from 4000-400 cm^−1^ (PC1: 34%, PC2: 23%, and PC3: 15%; Figure 4B) was smaller than the ranged data of 1500 to 1150 cm^−1^ (PC1: 81%, PC2: 10%, and PC3: 4%; Figure 4C). *Kappa* (*K*) identified by PCA-PLSR was observed by calibration:cross-validation with a 60:40 ratio for calibration:validation data using the 7 components, which reported a *K* of 0.52 and 0.81 and accuracy (Acc) of 0.67 and 0.88, respectively (Figure 4B,C).

### 2.4. Correlation with Principal Component Analysis (PCA)

**Figure 5:**
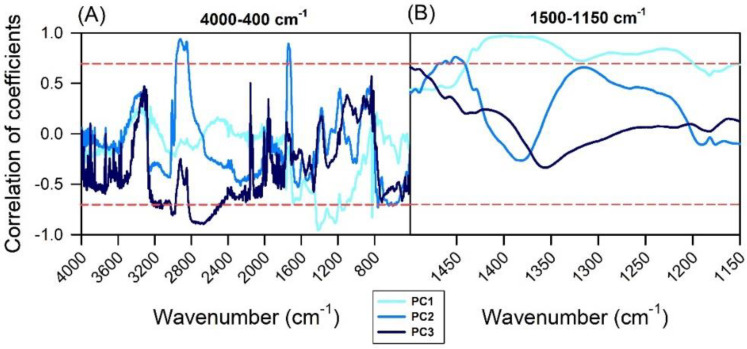
Correlation coefficients with the three principal components (light to dark blue lines, PC1, PC1, and PC3). (**A**) 4000-400 cm^−1^ by ATR-FTIR measurements. (**B**) 1500-1150 cm^−1^ by ATR-FTIR measurements. The red line indicates −0.70 and 0.70 correlation of coefficients.

The correlation between wavenumbers and PCs can be observed in Figure 5. The regions close to the 3600-3200 cm^−1^, 2800-2600 cm^−1^, 1800-1600 cm^−1^, 1500-1150 cm^−1^, 1100-800 cm^−1^, and 700-400 cm^−1^ vibrational bands presented the highest correlation values by functional pigment analyses. In this sense, for the first three principal components (PC1, PC2, and PC3), these regions may present properties and significant bands of differentiation among the varieties (Figure 5A). Consequently, the spectral bands of these regions can be selected to compose the linear discriminant analysis (LDA) and support vector machine classification (SVM)-based machine learning algorithms. In addition, the selected 1500-1150 cm^−1^ range showed a strong correlation for the discrimination of lettuce varieties based on vibration spectroscopy (Figure 5B).

### 2.5. Linear Discriminant Analysis (LDA) and Support Vector Machine (SVM) Based on Machine Learning Algorithms

**Figure 6:**
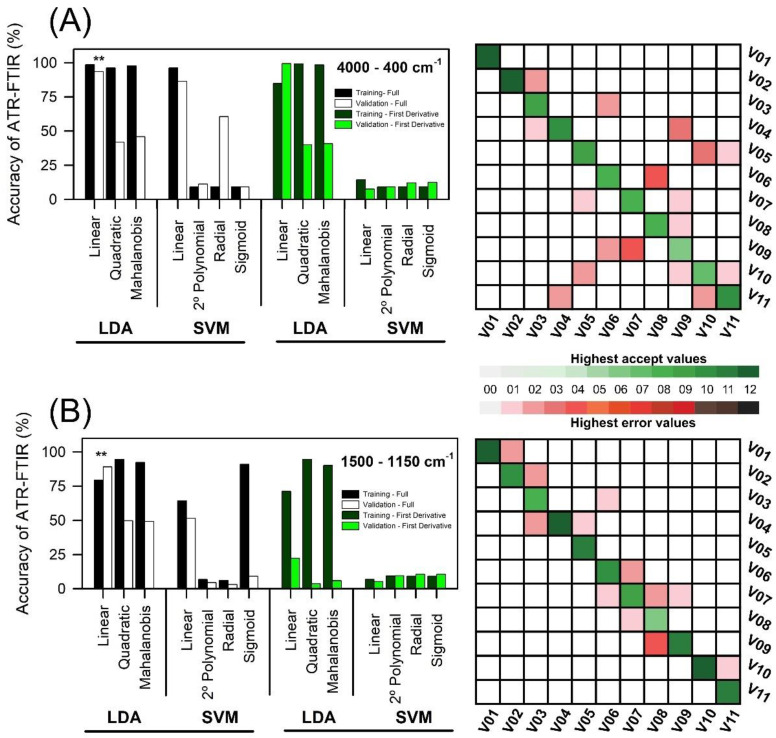
Accuracy of different machine learning algorithms based on linear discriminant analysis (LDA) and support vector machine classification (SVM) analyzed in full and transformed data (first derivative) on lettuce varieties ATR-FTIR spectroscopy data. (**A**) 4000 at 400 cm^−1^. (**B**) 1500 at 1150 cm^−1^. The first three PCA datasets were selected from the training and validation (testing) to create a confusion matrix for the machine learning algorithms, which showed higher overall accuracy. ** indicates the matrix confusion report on the right. The right boxes indicate the highest acceptance accuracy-precision, and the red boxes indicate the highest error accuracy-precision in the confusion matrix.

Machine learning algorithms were useful for sample classification by using ATR-FTIR spectroscopy raw data based on LDA and SVM. The overall accuracy was slightly enhanced using LDA-linear in relation to quadratic or Mahalanobis models or first derivate models using transformed data (Figure 6A,B). The first derivative spectra data did not show significant accuracy (highest error values or lower accuracy) (Figure 6A,B). In this sense, an overall accuracy of 98.6% in the 4000-400 cm^−1^ spectra and 79.5% in the 1500-1150 cm^−1^ range was obtained by the LDA-PLS method by using the first three PCs, carrying 95% data variance. The confusion matrix showed >97.9% accuracy to LDA-linear, contributing to lower misclassification between training and test data (i.e., 80 training samples and 52 test samples). Furthermore, 1500-1150 cm^−1^ ranged data showed higher accuracy in correctly classifying lettuce varieties, while full-spectrum machine learning algorithms did not classify lettuce varieties with similar precision. However, linear SVM showed 99.4% and 94.2% to quadratic-SVM in the training data, but having the lowest accuracy based on validation, 40.1% and 3.6%, respectively, of the data showed minor accuracy in classifying the lettuce varieties (Figure 6A,B; confusion matrix on the right).

Validation by LDA-PLS was carried out with the independent dataset, and the percentage of discrimination varied between 49.8 and 89.7%. Classification success was observed for all varieties based on machine learning algorithms. When range data (1500-1150 cm^−1^) were used, higher accept values with high accuracy and precision in the confusion matrix were found, although the full spectrum presented greater dispersion around the selection in the confusion matrix (Figure 6A,B; confusion matrix on the right).

The SVM analysis showed lower values (highest error values >37.69%) in relation to the LDA algorithms, even when using first derivative transformed data.

### 2.6. Prediction of Chemometric Parameters

PLSR models based on the calibration (Cal) and cross-validation (Cva) methods for biochemical and biomolecules related by pigment content parameters based on a vibrational band are shown in Table 2. Regarding ATR-FTIR spectroscopy data collected from 4000-400 cm^−1^ or 1500-1150 cm^−1^, a distinct difference in behavior was observed between the statistical stages (Cal and Cva). The best results (bold in Table 2) of R^2^_CV_ and RPD_CV_, Chl*a*, Chl*b*, Chl*a*+*b*, Car, AnC, Flv, and Phe to 4000-400 cm^−1^ and AnC, Flv, and Phe displayed very good and excellent prediction scores, respectively. Major values for R^2^_CV_ and RPD_CV_ metrics were obtained using full spectroscopy data for Phe, AnC, and Flv. In this sense, bias showed similar results (close to zero) for the calibration and cross-validation evaluated.

The prediction models for a pigment of the secondary metabolism present in lettuce parameters (i.e., biomolecules analyzed) were adjusted following the number of PLSR factors tested earlier by cross-validation (maximum of interaction but minor output noise). In this sense, the relationship between the predictor (wavenumbers) and predicted (lettuce chemometric parameters) indicated which variables were better explained using models containing the maximum of the seven factors for Chl*a*, Chl*b*, Chl*a*+*b*, Car, AnC, Flv, and Phe (Table 3).

The PLSR models, according to the R^2^_CV_, were considered very good (0.81–0.88) for Car, Anc, and Flv, and excellent (0.91–0.93) for Phe in the 4000-400 cm^−1^ data. Conforming to the RPD_P_ metric, six parameters were considered excellent (>2.10) in the models evaluated, with the exception of Chl*a* (Table 3).

The RMSE_CV_ of each variable was close to the RMSE_P_ (RMSE_CV_ ≈ RMSE_P_); for example, in some cases, it was slightly smaller, and slightly higher in others (Table 2 and Table 3). The values of bias were close to zero (AnC, and Phe, full spectra; AnC, and Phe, 1500-1150 cm^−1^) or negative for many (Chl*b*, Chl*a*+*b*, Car, 4000-400 cm^−1^; Chl*a*, Chl*b*, Chl*a*+*b*, Car; 1500-1150 cm^−1^) variables in the cross-validation and prediction analyses.

All seven evaluated parameters were adjusted with an independent dataset (ATR-FTIR spectroscopy data) from those used in the cross-validation phase to evaluate the ability of PLSR models to predict these chemometric parameters. In this sense, the reference vs. predicted data, as well as the results of the multivariate statistical results and machine learning algorithms, are shown in Table 2 and Table 3.

The models of the predicted lettuce variety parameters were adjusted according to PLSR of the number based on factors before being tested by cross-validation phases. Therefore, the relationship between the predictor (wavenumbers; vibrational bands) and predicted (chemometric; lettuce parameters) variables was better explained using models containing five or seven factors for 1500-1150 cm^−1^ and 4000-400 cm^−1^, respectively.

### 2.7. Regression Coefficients (RCs) and Variable Importance in Projection (VIP)

**Figure 7:**
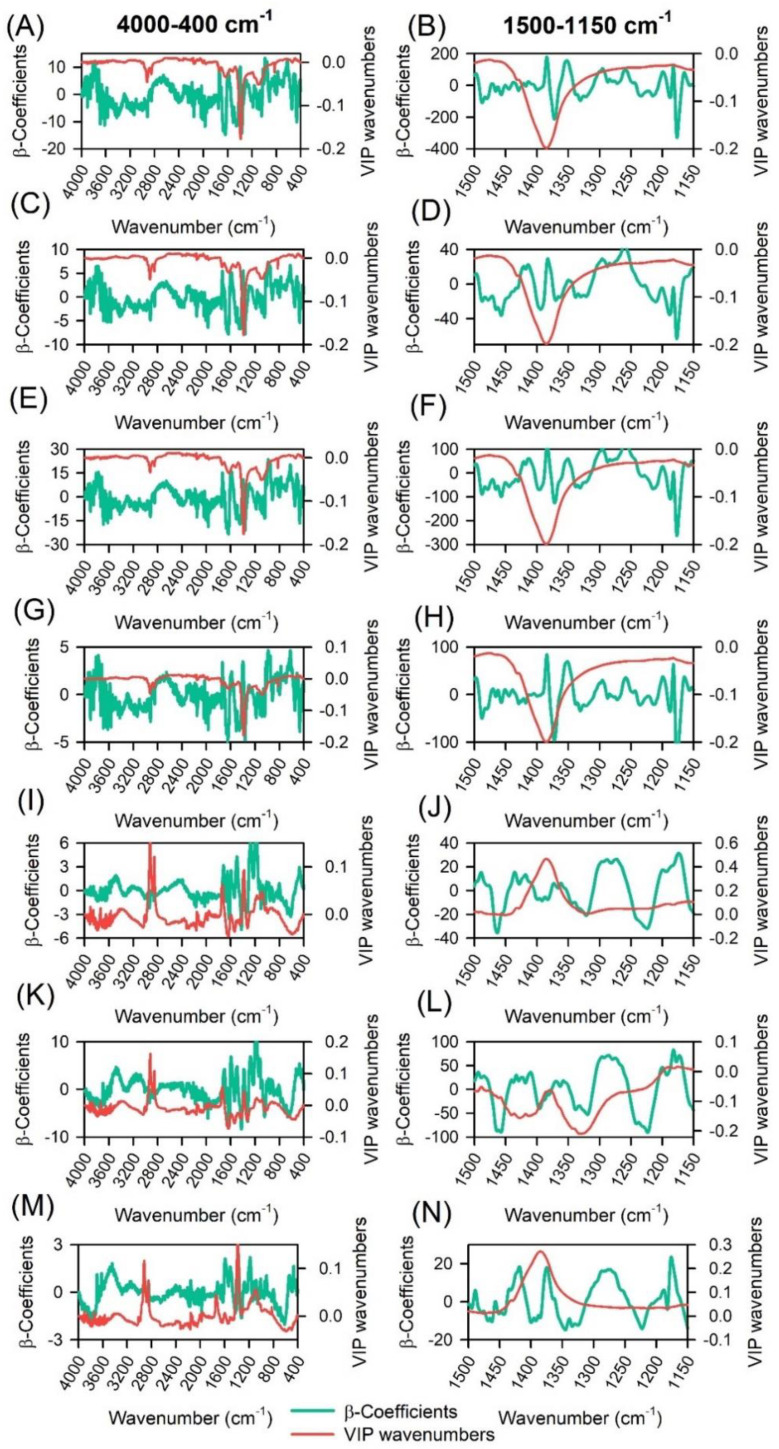
β-Coefficients (RCs) and variable importance to the projection (VIP) method-based ATR-FTIR spectroscopy. (**A**,**C**,**E**,**G**,**I**,**K**,**M**) to 4000 at 400 cm^−1^ and (**B**,**D**,**F**,**H**,**J**,**L**,**N**) to 1500 at 1150 cm^−1^ by ATR-FTIR spectroscopy. (**A**,**B**) Chlorophyll *a* (mg m^−2^); (**C**,**D**) Chlorophyll *b* (mg m^−2^); (**E**,**F**) Chlorophyll *a* + *b* (mg m^−2^); (**G**,**H**) Carotenoids (mg m^−2^); (**I**,**J**) Anthocyanins (nmol m^−2^); (**K**,**L**) Flavonoids (nmol m^−2^); (**M**,**N**) Phenolic compounds (mL L^−1^).

The β-coefficients (RCs) of the regression and variable importance in projection (VIP) metrics of the PLSR model are reported in Figure 7. Many regions of valleys and peaks where the RC and VIP applied a substantial effect on the construction of the prediction model were generally well distributed among all spectra or ranged wavenumbers (medium IR-bands).

The RC and VIP values used for PLSR models contrast between 10 and 16 selected wavenumbers (peaks and valleys). In some cases, the same wavenumbers were selected using 4000-400 cm^−1^ or 1500-1150 cm^−1^ data (Table 4; Figure 7). In this sense, the parameters that obtained RPDs higher than 3.00 (Table 3) (AnC, Flv, and Phe) found excellent predictions for VIP-selected wavenumbers to improve models (Table 4).

The fingerprints of ATR-FTIR spectra in Table 5 could be simplified by using the most VIP values of wavenumbers observed in the majority 1500-1150 cm^−1^. In addition, many values between 1800-1500 cm^−1^ were related to phenolic compounds. Thus, values in reference bands vs. experimental sample spectra showed a high correlation between the pigments analyzed. For example, anthocyanin and flavonoid fingerprints were marked by ring stretching of C=C or aromatic rings to vibrational bands. VIP showed many bands related to aromatic stretching ν(C=C), stretching ν(C–C), or ring stretching –(C=O), which could be Phe, Flv, as well as –CH stretching by AnC, Chls, and Car compounds (Table 4 and Table 5).

## 3. Discussion

### 3.1. Descriptive Analysis and Cluster Heatmap

The variability observed in lettuce varieties with distinct pigment contents (Figure 1 and Figure 3) was explored to estimate parameters such as Chl*a*, Chl*b*, Chl*a*+*b*, Car, AnC, Flv, and Phe based on the ATR-FTIR spectra between 4000-400 cm^−1^ (Figure 2 and Figure 3). Some approaches used (Table 3 and Table 4; Figure 2, Figure 3, Figure 4, Figure 5 and Figure 6) aid a rapid determination with high-throughput measurements that produce data-rich results. In particular, ATR-FTIR fingerprint-based biomolecule analysis techniques should play a key role in the development of fast, efficient, and simple prediction crop phenotyping in response to monitoring the dynamic bioaccumulation of pigments [7,9,28,35]. However, some statistical approaches, combined with machine learning algorithms (LDA and SVM), were labeled as having low and moderate prediction accuracy when the first derivative or 2° polynomial, radial, or sigmoid was applied with algorithms (Table 2 and Table 3; Figure 4 and Figure 6).

In general, comparison of the full-green, green-purplish, and full-purple groups showed that plants were associated with high plastic development and high pigment content. Natural variability interferes with machine learning algorithms or classification based on cluster analyses (Figure 1 and Figure 3). In this sense, the cluster heatmap showed the highest efficiency in grouping similar lettuce varieties based on pigment profile analysis [48,49,50]. Heatmaps are a better tool to differentiate varieties or distinguish possible interactions of molecules present in leaves [51,52]. For example, a frequent collection of data from different leaves accumulates distinct levels of pigments. Under these conditions, it was possible to separate large differences between the minimum and maximum values observed with high accuracy from ATR-FTIR [14,22,42]. We observed that the relationship among pigments was more significant than the absolute contents. Additionally, it is possible to associate many tools to classify plants based on ATR-FTIR to understand the clustering of lettuce varieties [53,54] and other crops [55,56].

### 3.2. ATR-FTIR Spectroscopy and Tracking of Fingerprints

The differences among varieties were detected in the all-spectroscopy curve [42]. All spectral curves exhibit bands associated with vibrational modes from pigments, such as carotenoids and chlorophylls, which are more or less intensely detected, and those variations are related to pigments in each lettuce variety. Many compounds and proteins present in leaves contribute to the total vibration bands. However, the absorbance of a chemical band with characteristic vibrations defines functional groups, such as Flv or AnC, that decrease in green lettuce varieties [25,57]. Those outside chloroplast pigments from minor vibrational bands could be related to pigments present in vacuoles. Flv and AnC were often present at higher levels in the leaf epidermis [9,48] (Table 5). At 1800-1500 cm^−1^, the highest values were related to the vibrational wavenumber related to the cell wall components but not the chloroplast pigments.

Concerning the chlorophyll variable (Figure 4), the high RC and VIP values at 3467-3353 and 1732-1151 cm^−1^, which are related vibrational bands, possibly obtained a higher precision and were more stable in the characteristics of each lettuce variety. The value obtained at 1764-1658 or 1550-1443 cm^−1^ is associated with the maximal and minimum reflectance by chlorophyll. Notably, the absorption of 1550-1150 cm^−1^ was influenced by a few bands by the chlorophyll content [58] to distinguish between green-purple varieties. In addition, the chlorophyll content and these vibrational bands promote biomass accumulation in plants and promote possible predictions of these pigments [3,22,36]. The bands at 2800-2500 and 1200-1100 cm^−1^, in turn, are possibly due to the constituent by amino acids and proteins (for example, N–H in plane banding/C=O stretch + N–H bend/C–N stretch combination bands) in plant constituents and metabolites, which could likewise be associated with many important dissipative energies (carotenoids) and antioxidant molecules that protect lettuce [3,17,58,59,60,61].

At 1750-1700 cm^−1^, substantial differences were detected in the spectral analysis. Spectral data in this region support obtaining fingerprints related to vibrational modes of phenol compounds and aromatic groups, such as in 1750-1735 cm^−1^ associated with saturated aliphatic esters (C=O stretching), 1740-1720 cm^−1^ associated with saturated α,β-unsaturated esters and aliphatic aldehydes (C–C) or 1715 cm^−1^, followed by saturated aliphatic ketones [14,16,42]. In addition, the peak at 1735 cm^−1^ was characterized by double bond stretching (νC=O) from ester bonds, which may be linked to lignin in cell walls or other phenolic compounds deposited on cells and soluble phenolics [62]. Other research suggests that the peak at 1700 cm^−1^ was similar to the keto C=O band of chlorophylls [46,63]. The peak at 1670 cm^−1^ by FTIR showed good correlation with the stretching vibration of carbonyl (C=O) and amide I (C–N). Furthermore, the peak at 1640-1630 cm^−1^ can be correlated to the stretching of aromatic C=C bonds in anthocyanins and other phenolic compounds with chlorogenic acids, coumarins, catechins, flavonoids, and antioxidant compounds [7,42,64].

Moreover, bands at 1660, 1650, 1606, 1545, and 1515 cm^−1^ at a stretching of C=O referred to the amide I and II groups, and aromatic elongation C=O was linked to flavonoids, anthocyanins, and other soluble phenolics. Additionally, it was reported [7,25] that an elongation of C=N and NH, referring to amide II, and a stretching of aromatic C=C, mainly indicated phenolic compounds. In addition, the 1800-1501 cm^−1^ spectral range included many wavenumbers corresponding to spectral double bond (C=C) stretching (Table 4 and Table 5; Figure 4). It is possible to link it to anthocyanins and flavonoids [11,14,41]. The peak at 1640-1630 cm^−1^ can be correlated to the stretching of aromatic C=C bonds in anthocyanin molecules [25]. Specifically, 1640 cm^−1^, following the report in [48], was related to the vibrational modes of C=C groups in aromatic rings. The 1440 to 1436 cm^−1^ peak corresponds to vibration aromatic stretching (νC=O) and double bond stretching between carbon (νC=C). Furthermore, the region between 1260 and 1180 cm^−1^ is known to be related to the phenol compound vibrational mode due to stretching out of the C–C–O phase. For example, peaks between 1293 and 790-760 cm^−1^ were associated with flavonoids, such as O–H and aromatic ring vibrations. Following the report in [45], a wavenumber of 1640 cm^−1^ was correlated, in which these bands were mainly occupied by vibrational motions of C=C groups in the aromatic rings. In this sense, other research has similarly reported evidence for flavonoids and phenolic compounds when using hyperspectroscopy, ^1^H-NMR/MRS spectroscopy, and analysis by multivariate tools [53]. Additionally, peaks before 1410 cm^−1^ were reported for phenolic compounds, even at 1663 cm^−1^ for possible terpenes as C–H bonds attached to the benzene ring of phenol and aromatic molecules. All peaks contribute to the elucidation of lettuce ATR-FTIR fingerprints [9,11,65,66,67].

The range of 1455-1440 cm^−1^ is associated with –CH_2_ and –CH_3_ asymmetric bending related to polysaccharides, lipids, and proteins present in plant samples. In addition, 1430 to 1420 cm^−1^ is associated with –OH bending with polysaccharides, alcohol, and carboxylic acid in the cell wall. The bands at 1440 to 1436 cm^−1^ were associated with phenolic compounds due to the vibrational mode of aromatic stretching between carbons (νC–C) conjugated with a double bond elongation between carbons (νC=C) [24,25]. The region between 1260 and 1180 cm^−1^ is known for the vibrational mode of phenols due to out-of-phase C–C–O stretching, which can be associated with flavonoids since they belong to the group of phenolic compounds [9,48]. In this sense, the 1197 cm^−1^ peak likely corresponds to the band/peak of anthocyanin molecules (r = 0.960; *p* < 0.001), and similarly, the following results are shown in [25]. Here, ATR-FTIR data also showed a high and positive correlation for anthocyanins and the 1197 cm^−1^ peak (Table 4 and Table 5; Figure 3 and Figure 7) [53,62,68], followed by analyses by Gaussians or LDA algorithms (Figure 3 and Figure 4). ATR-FTIR spectroscopy displayed stretching C–O modes that are mainly assigned to the region from 1198 to 1025 cm^−1^ for symmetric elongation of the C–O–C group in the pyran ring of condensed tannins and other flavonoids and phenolic compounds [10,22], similar to the report in Figure 4, Figure 6 and Figure 7. For example, for anthocyanins, flavonoids, or phenolic compounds, the corresponding asymmetric stretching occurs approximately in the 1285 to 1260 cm^−1^ spectral range [69,70]. Analogously, the 1202 to 1200 cm^−1^ peak, attributed to –C–OH aliphatic stretching, is reported to be a typical feature for red wine or purple carrots correlated with condensed tannins [22] or increased anthocyanin levels (Figure 3). Thus, the data obtained by PCA and LDA proved to be good tools by which to discriminate the most significant spectral bands following [14,15,22,25,41].

When all spectroscopy curves were applied to distinguish a particular variable, only some FTIR-specific bands were used, and the effects of several background errors on misclassification were reduced, as described in [17,36,71]. In this sense, when applied, the full spectrum might improve the accuracy and reliability of the analysis to better generate a prediction model. Similarly, [72] reported that the effects of various external-factor interference sources can be reduced or eliminated when using the total curve in relation to using individual or only a few peaks [59,73]. In general, the full spectrum, in contrast to a specific range, is more appropriate, following [17,36,71], which demonstrated a valuable method to analyze ATR-FTIR by LDA and SVM, with greater accuracy and precision to classify and predict functional pigment content in lettuce varieties.

### 3.3. Efficiency of Machine Learning Algorithms to Classify Lettuce Plants

Machine learning algorithms were applied to sample group classification by using ATR-FTIR spectroscopy raw data and PCA, LDA, and SVM machine learning algorithms. As a whole, the accuracy was slightly improved by using PCA and LDA data. Using ranged data (1500-1150 cm^−1^) produced superior accuracy in relation to the full spectra (4000-400 cm^−1^), which showed higher differences in the overall accuracy between datasets. Using the first three PCs improved computational processing because they contained a reduced dimension. An overall accuracy of >92.2% for linear LDA and SVM was obtained by using three PCs with ≈95% of variance. Similarly, but with slightly higher accuracy at 1500-1150 cm^−1^ in the ATR-FTIR spectroscopy method, LDA-linear carried 95.6% of the data variance. Following [11], data mining and machine learning can be improved to classify with higher precision in relation to quadratic, Mahalanobis, or 2° polynomial, radial or sigmoid validation methods (testing), and SVM or LDA can be considered the most promising algorithm, since it reached an accuracy with the smallest number of PCs (Figure 6). However, this is true for all machine learning experiment analyses of experimental data following [11,14,74]. The influence of data distribution was more significant for machine learning (based on a linear interaction-pigment contents vs. lettuce varieties), which possibly contributes to the high accuracy and separation of the analyzed lettuce groups [3,53,54].

Many studies use PCA to extract the most useful FTIR or ^1^H-NMR spectroscopy information [7,14,75], which is a useful method to classify and discriminate the most responsive wavenumbers [14,43,67]. This method shows good results in situations with fewer impurities (high signal-to-noise ratio) or simple samples [14,59]. For lettuce, there are uncountable types of molecules present in leaf powder that affect the results, and which produce a more complex spectrum. There are many interferences in a particular band. Choosing appropriate bands from all available bands is a challenge to obtain useful information. It often requires expert knowledge and rich practical expertise, and it is not easy to achieve automated and rapid detection. SVM and LDA are often used for the quantitative analysis of spectra, but LDA adjusts many features when processing spectroscopy data, which leads to the occurrence of overfitting (Figure 2 and Figure 6).

### 3.4. Partial Least Squares Regression (PLSR) for Classification-Prediction Pigments

The results of R^2^_CV_, R^2^_P_, RPD_CV_, RPD_P_, RMSE_CV_, and RMSE_P_ for the full (4000-400 cm^−1^) and selected spectra (1500-1500 cm^−1^) were modified according to the parameters estimated (Table 2; Figure 4). As a whole, the approach used (Figure 6) showed good results. Other techniques and multivariate statistical analysis might achieve similar predictive results using variable importance to the projection (VIP), STEPDisc tools, regression coefficients (RCs), STEPWISE methods, or processes with noise reduction efficiency. In this sense, standard normal variate (SNV), Savitzky–Golay (SG), multiplicative scatter correction (MSC), and other correction methods vary according to the attribute measured but have been alternatively used to obtain good to excellent results based on PLSR methods [5,13,24,53]. It is interesting and very important because pathways of secondary metabolism produce many biomolecules in the plant cell. For example, the inclusion of vibrational modes induced by spectroscopy, such as –NH_3_, –N–H, and –C–H stretching vibrations from aromatic rings associated with specific wavenumbers and associated with compounds along with the structure of the pigments, proteins, and fatty acids, allowed us to obtain better PLSR models with statistical metrics, such as R^2^ and RPD, ranging from good to excellent, respectively, for all assessed chemometric vibrations [4,35,42,54].

Cross-validation showed that the results for the statistical metrics were slightly higher than those obtained in the prediction phase, as projected, since the number of samples used to obtain the model was smaller in the calibration phase [76]. Moreover, [71,76] used ATR-FTIR spectroscopy methods to predict distinguishing pigments in lettuce varieties and similarly obtained an increase in RMSE in the prediction phase, which connected many metabolites produced in secondary metabolism [17].

However, in the prediction phase, the R^2^_P_ values calculated for Chl*a*, Chl*b*, Chl*a*+*b*, Car, AnC, Flv, and Phe were similar to other variables from red lettuces [4], green tea [46], *Camellia sinensis* [77], *Enhalus acoroides* [16], tobacco [17], and peatlands [78] employing SWIR and/or MWIR and/or LWIR spectroscopy or hyperspectroscopy [14,79,80,81,82]. In this research, the difficulty of establishing metrics with better results for the leaf pigments (chloroplast and outside-chloroplast pigments, particularly in the phase of the prediction) could be due to the limited number of samples (*n* = 52). In addition, the authors of a previous study [36] also used specific range bands and were able to define R^2^_P_ with better metrics [36,71], but RPD, offset, or bias obtained the lowest precision. However, when they set specific spectral peaks or valleys, the prediction models showed high precision, but with higher noise by the backgrounds and lower accuracy [4,5,55].

Our data showed the high accuracy of the model to estimate attributes with spectroscopy techniques confirmed to be useful (or excellent according to the Offset and RPD_P_) for many of the variables (i.e., biomolecules) tested. In this sense, the main advantage of the proposed method is the ability to simply form by predicting simultaneous inferences from seven chemometrics (Chl*a*, Chl*b*, Chl*a*+*b*, Car, AnC, Flv, and Phe). In this sense, it could be used for monitoring many molecules and metabolites synthetized by secondary metabolism. In addition, the crop production status in a single collection without the need for preparation using reagents or expensive preparation of samples (i.e., Si and KBr) and high-cost equipment for acquisition (^1^H-NMR, LC-MS/MS, SAXS, DRX, or hyperspectroradiometer) [2,17,71].

### 3.5. Regression Coefficients

Regression coefficients (RCs) and variable importance in projection (VIP) were demonstrated with essential tools to classify and avoid possible errors to estimate by correlations. Thus, this work aimed to better understand how each variable (wavenumbers) contributed to the significant variation in the model used to estimate the parameters. In most cases, high RC (Figure 4 and Figure 7) and VIP wavenumbers (Table 4; Figure 7) were well distributed across the entire portion of the spectrum for all groups of molecules analyzed [14,83].

Many studies have investigated the potential of physical tools as a technique for estimating radish, cauliflower, lettuce, native species, and other important crop and cultivate species, which, by pigments, primarily consider the region with wavenumbers less than 1500-1150 cm^−1^ [34,35,53]. In general, they were biased and obtained low-accuracy outputs. In the present study, higher regression coefficients were obtained using full range values. We highlight that, on average, 23.4% of VIP values were shared with ranged data; in other words, ≈75% of VIP values were selected outside of the selected values.

Acquisition of full spectra using spectroscopy sensors and analysis by curve deconvolution associated with PLSR [84] and many other multivariate tools [85,86,87] show supplementary, robust, and reliable models, as evidenced by the RMSE values that are related to the use of range bands. The use of full bands takes advantage of specific wavenumbers [2,12]. In this sense, the flexibility of choosing between different spectral bands by performing a discriminant analysis linked with the high precision and accuracy of high-resolution spectral data enables metabolites to be identified in secondary metabolism, variety discrimination, predicted pigment content and concentrations, and applied machine learning in ATR-FTIR spectroscopy tools [3,12,36,53].

### 3.6. Benefits and Limitations of Using ATR-FTIR Spectroscopy for Classified and Predicted Pigments in Lettuce

The method used here with available spectroscopy allows us to rapidly and precisely categorize based on pigment and vibrational group profiles. In addition, the capacity to specifically discriminate between variety-mediated regulation of development and dynamics is applicable to monitoring crops and dynamics of the metabolite states in lettuce plants [4,7,22,36,58]. By combining ATR-FTIR spectroscopy data and robust multivariate statistical modeling-based machine learning, this research described a prospective method with the possibility of concomitantly classifying and predicting the spectroscopy pigments in leaves.

Furthermore, the knowledge of wavenumbers for FTIR spectroscopy phenolic/anthocyanin compound analysis variability among lettuce varieties could be used at a commercial level as increasingly important criteria for the identification of new high-performing genotypes and cultivars for particular environments and cultivation conditions [2,86], which are rich in phytochemicals and have potential health benefits. In this sense, agronomic protection, food security, and molecular characterization showed the highest accuracy of ATR-FTIR tools, together with machine learning algorithms, in making better decisions regarding increased productivity and selecting the most responsive varieties in biomolecules plant and crop sciences.

Many metabolites (molecules, biocompounds, or pathways of secondary metabolism) differentiate varieties of crop plants. However, their differentiation is challenging (i.e., depending on the plant genotype or species), and currently, still depends on human expertise. In this sense, they are associated with many molecules, including chloroplast or outside-chloroplast molecules [11,13,35].

Finally, techniques involving ATR-FTIR spectroscopy, together with machine learning algorithm approaches, are highly valuable and promising in regard to meeting this need, as they are fast and do not require previous sample preparation with chemical reagents (Si and KBr), or expensive and classic equipment [2,71]. Thus, in this study, we showed that the use of ATR-FTIR spectra is a promising strategy for understanding, classifying, and predicting molecular content relationships derived from eleven lettuce varieties, and could be applicable to crop plants with satisfactory accuracy and precision.

## 4. Material and Methods

### 4.1. Plant Material, Growth Conditions, and Experimental Design

Lettuce seeds (*Lactuca sativa* L.) were germinated on Germitest^®^ paper immersed in 4 mL of Hoagland’s solution (pH 5.4) in a dish. After 15 days of growth, seedlings were transplanted to MecPlant^®^ (MecPrec Ind., Telêmaco Borba, Paraná, Brazil), a commercial substrate, and then transported to grow in a greenhouse.

Experiments were conducted in the greenhouse at the State University of Maringá, Maringá, Paraná, Brazil. Eleven lettuce varieties (V_X_) were grown (V01—Rainha de Maio, V02—Vitória, V03—Maravilha de Inverno, V04—Grandes Lagos Americana, V05—Mimosa Prado, V06—Quatro Estações, V07—Batávia Joaquina, V08—Mimosa Vermelha, V09—Batávia Cacimba, V10—Pipa and V11—Mimosa Rubi) in pots with a capacity of 3 L containing substrate (soil:sand:organic compounds, in a proportion of 3:2:2). Fertilization was applied with N-P-K (10-10-10) as recommended for lettuce plants. Light was monitored and reached 1200 µmol m^−2^ s^−1^ irradiance, measured by a LI-190R quantum sensor (Li-Cor Inc., Lincoln, NE, USA) under a natural photoperiod (approx. 12/12; light/dark) at 30 °C (±5 °C) with 50–80% relative humidity. The varieties were visually chosen by color (green to purple colors) due to the classes and content of different pigments (Figure 1). The cultivation was conducted through a completely randomized design with 11 treatments and 12 biological repetitions.

### 4.2. Leaf Analysis

Leaf area was measured using an LI-3100C leaf area meter (Li-Cor Inc., Lincoln, NE, USA) and was used to estimate the leaf pigment content expressed in area.

### 4.3. Leaf Pigment Profiling

Simultaneous quantification of chlorophyll *a* (Chl*a*), chlorophyll *b* (Chl*b*), total chlorophyll (Chl*a*+*b*), carotenoids (Car), anthocyanins (AnC), and flavonoids (Flv) in methanolic extracts of the leaves was conducted, as described in [21,88]. Spectral scans were performed on a Lambda 1050 UV/VIS/NIR Spectrophotometer (PerkinElmer, Inc., Waltham, MA, USA). The Chls, Car, AnC, and Flv concentrations were reported based on area unity.

### 4.4. Total Soluble Phenolic Compound Quantification

Total soluble phenol (PhC) quantification was carried out exactly as described in [2,3] and analyzed on a Lambda 1050 UV/VIS/NIR Spectrophotometer (PerkinElmer, Inc., Waltham, MA, USA) at 725 nm. The equivalent PhC concentration was determined using gallic acid as a reference, Y = 87.65x + 1.651; r^2^ = 0.994.

### 4.5. Attenuated Total Reflectance Fourier Transform Infrared Spectroscopy (ATR-FTIR) Analysis

Fourier transform infrared spectroscopy was performed on oven-dried (70 °C) leaf samples with a Bruker Vertex 70v FTIR spectrometer (Bruker Optik GmbH, Ettlingen, Germany, DEU) equipped with A225/Q Platinum attenuated total reflectance (ATR) with a single reflection diamond crystal (Bruker Optik GmbH, Rosenheim, Germany, DEU). The spectra were obtained from 4000 to 400 cm^−1^ wavenumbers with a spectral resolution of 4 cm^−1^. Two replications were performed for each spectrum, which were collected from an average of 300 scans sample^−1^ at room temperature (25 °C). All spectra were equally corrected by baseline using OPUS software (Bruker Optik GmbH, Rosenheim, DEU), and the intensity was normalized at 3270 cm^−1^ (OH stretching band).

### 4.6. Statistical Analyses

#### 4.6.1. Descriptive Analysis

Statistical analyses for the classification and prediction of pigment contents were performed using The Unscramber x10.4^®^ (Camo Software, Oslo, Norway, NOK), SigmaPlot^®^ 10.0 software (Systat Software Inc., GmbH, Ettlingen, Germany, DEU), Statistica^®^ 12.0 software (Statsoft Inc., Tulsa, OK, USA), Excel 2021^®^ (Microsoft Office Inc., Sunnyvale, CA, USA), and the R package R-Core-Team (2020) (https://www.R-project.org (accessed on 20 September 2022)) [87].

#### 4.6.2. Statistical Analyses of the ATR-FTIR Spectral Signature

The effects of ATR-FTIR spectra on the untransformed wavenumber profiles (averaged per sample). Data were evaluated using PERMANOVA by using Euclidian measurement algorithms of dissimilarity using the Euclidean distance with the “vegan” package in R-Core-Team (2020) (https://www.R-project.org (accessed on 20 September 2022)).

#### 4.6.3. Principal Component Analysis (PCA)

Principal component analysis (PCA) was performed using “The Unscrambler X” software, version x10.4 (CAMO AS, Oslo, Norway, NOK), as an indicator of whether the variance in the ATR-FTIR spectroscopy between the lettuces could be explained or not (*p* < 0.05) and how effectively biomolecules based on secondary metabolism in plant varieties could be clustered. The numbers of principal components (PCs) were based on the highest average *Kappa* (*K*) and accuracy (Acc) values obtained for the validation models from partial least squares regression (PLSR). PLSR analysis was performed exactly as described in [2,3,17].

#### 4.6.4. Linear Discriminant Analysis (LDA) for Machine Learning

Linear discriminant analysis (LDA) was used to obtain models to classify each ATR-FTIR spectroscopy-based machine learning algorithm of lettuce variety plants. Before obtaining the discriminant models, a routine PCA-LDA (three components) was performed to select wavenumbers that best explained the differences in lettuce varieties [84,87]. The routine PCA-LDA selects the variables to compose the model one by one, in accordance with the partial F value input in each stage, the Wilks’ lambda value, and partial variables, such as R^2^. The proc LDA technique is carried out until no variable can be arrived at or removed from the model, as described in [2,3,17].

The ability and quality to acceptably classify the obtained models of the LDA were evaluated based on external data for the validation method by random dataset division (60:40), as described in [3]. Linear, quadratic, and Mahalanobis models of analysis were applied to classify lettuce-based machine learning algorithms [50].

#### 4.6.5. Support Vector Machine (SVM) for Machine Learning

The support vector machine (SVM) is a supervised, kernel-based learning method [11,13,33]. Using kernel functions and a first derivative, SVM applied to a spectroscopy dataset allows for higher-dimensional feature space data than the original ATR-FTIR spectroscopy, as described in [11,13,35,89]. In addition, the k-kernel reports should be viewed as a mapping of nonlinear data to a higher dimensional feature space while providing a computation short-cut by allowing linear algorithms to work with a higher dimensional feature space [11,13,35,89]. The last decision-based purpose of the SVM is determined by a few support vectors (machine learning). The function based on a complexity algorithm depends on the number of support vectors based on 4000 to 400 cm^−1^ and 1500 to 1150 cm^−1^. The training and validation (testing) of the SVM algorithms were performed to obtain the accuracy of ATR-FTIR data analysis to predict contents similar to chemical analysis.

#### 4.6.6. Partial Least Squares Regression (PLSR) Analysis of ATR-FTIR Spectroscopy

The data were subjected to Shapiro–Wilk and Bartlett’s tests to evaluate the normal distribution, homogeneity, and variance before obtaining the prediction models of quality (chlorophylls, carotenoids, anthocyanins, flavonoids, and phenolic compounds). For all variables, 4000 to 400 cm^−1^ or 1500 to 1150 cm^−1^ were recorded to improve the accuracy of the data. Subsequently, the data (ATR-FTIR spectroscopy of lettuce varieties) were centered on the mean and subjected to PLSR analysis. The algorithm for model inputs was NIPALS, and output outlier limits were defined by Leverage’s type and analyzed using Hotelling’s T^2^ test (limit of 5%). Spectral data of the 132 samples of different secondary metabolites collected were divided into two groups (based on biological replicates). The first group consisted of 60% (80) of the samples with the aim of generating the model (training), while the second group was represented by 40% (52) of the remaining samples with the aim of testing (prediction) the PLSR model, exactly as described in [2].

The calibration (Cal) and leave-one-out cross-validation (Cva) methods were used to classify and predict the quality parameters based on chemometrics [biochemical compounds, i.e., chlorophyll *a* (mg m^−2^), chlorophyll *b* (mg m^−2^), chlorophyll *a*+*b* (mg m^−2^), carotenoids (carotenes and xanthophyll; mg m^−2^), anthocyanins (nmol m^−2^), flavonoids (nmol m^−2^), and phenolic compounds (mL L^−1^)].

The predictive capacity of the calibration models was estimated by calculating all metrics, such as R^2^ (coefficient of determination), offset, RMSE (root mean square error), and RPD (ratio of performance to deviation), and bias was determined to assess the quality and accuracy of the model, exactly as described in [3,8,17,71]. The simplified flowchart is displayed in Figure 2.

## 5. Conclusions

As shown in the present study, each lettuce variety has unique spectral signatures of secondary metabolites based on ATR-FTIR spectroscopy. Their relationship, based on a heatmap, is a promising tool for clustering varieties. A typical spectral signature, even as attributed to vibrational excitation of –C–H bonds, covalently bonded (νC=O), (C=O stretching), aromatic C=C, –C–H, –N–H, –NH_3_, –COOH, –O–H stretching from aromatic rings, is associated with the main compounds present in lettuce varieties. All pigment-based chemometric analyses, such as Chl*a*, Chl*b*, Chl*a*+*b*, Car, AnC, Flv, and Phe, were efficiently estimated and categorized using ATR-FTIR spectroscopy together with linear discriminant analysis (LDA) and principal component analysis (PCA)-based machine learning algorithms.

The estimated PLSR models presented R^2^_P_ and RPD_P_ values > 0.80 and >2.10, respectively, for many variables predicted based on the full range (4000-400 cm^−1^) or selected range (1500-1150 cm^−1^) from ATR-FTIR spectra. The most important wavenumbers were well distributed within the three operating ranges, generally close to 3000-2700 cm^−1^, 2600-2200 cm^−1^, 1700-1500 cm^−1^, 1400-1150 cm^−1^, and 650-550 cm^−1^, as highlighted by the regression coefficients.

Thus, our main objective was achieved in the present study, which shows the potential of ATR-FTIR spectroscopy coupled to multivariate analysis to classify and estimate specific metabolites in lettuce plants. Therefore, this procedure is a promising alternative, as it offers the advantages of being rapid and fast, and does not require previous sample preparation (i.e., Si and KBr by chemical reagents) and accurate data acquisition over a wide infrared range. ATR-FTIR spectra analyzed by machine learning algorithms could facilitate data processing. Their potential can be applied to field or other metabolites based on their vibrational bands, with an excellent capacity for monitoring and machine learning applied in lettuce and possibly to other crop-field plants.

## Figures and Tables

**Table 1:** Descriptive analysis in lettuce plants. Parameters: Chlorophyll *a* (Chl*a*; mg m^−2^); Chlorophyll *b* (Chl*b*; mg m^−2^); Chlorophyll *a* + *b* (Chl*a*+*b*; mg m^−2^); Carotenoids (Car; mg m^−2^); Anthocyanins (AnC; nmol m^−2^); Flavonoids (Flv; nmol m^−2^); Phenolic compounds (Phe; ml L^−1^).

Parameters	Count (*n*)	Mean	Median	Minimum	Maximum	CV (%)
Chl*a* (mg m^−2^)	132	128.89	118.95	63.15	306.40	39.18
Chl*b* (mg m^−2^)	132	66.76	61.75	39.41	147.27	35.09
Chl*a*+*b* (mg m^−2^)	132	195.65	182.51	107.96	453.67	37.64
Car (mg m^−2^)	132	45.70	42.41	25.56	101.92	35.47
AnC (nmol m^−2^)	132	13.43	11.46	0.00	68.57	126.53
Flv (nmol m^−2^)	132	59.55	49.75	11.77	178.28	60.66
Phe (mL L^−1^)	132	62.59	66.20	42.74	78.66	16.79

**Table 2:** PLSR model in calibration and cross-validation at wavenumbers of 4000 at 400 cm^−1^ and 1500 at 1150 cm^−1^. Model goodness-of-fit (R^2^), offset, root mean square error (RMSE), and ratio of performance to deviation (RPD) for calibration (Cal) and cross-validation (Cva) data generated using 132 random permutations of the data with 60% used for Cal and 40% used for Val for the PLSR models predicting parameters biochemical pigments (chemometrics) contents from ATR-FTIR spectroscopy of lettuce leaves. Bias outputs are not shown, as they were always lower than 0.01 for both Cal and Cva. The bold represents significant regression models (R^2^). The underline indicates a bad residual to the prediction deviation (RPD). Parameter abbreviations—see Table 1 or Abbreviation list.

Wavenumbers	Parameters	Calibration				Cross-Validation			
		R^2^	Offset	RMSE	RPD	R^2^	Offset	RMSE	RPD
4000-400 cm^−1^	Chl*a* (mg m^−2^)	**0.77**	31.70	24.91	2.09	**0.76**	33.63	26.60	2.04
	Chl*b* (mg m^−2^)	**0.78**	14.35	10.93	2.13	**0.75**	15.20	11.64	2.00
	Chl*a*+*b* (mg m^−2^)	**0.79**	41.80	33.73	2.18	**0.76**	44.58	36.03	2.04
	Car (mg m^−2^)	**0.81**	8.55	7.05	2.29	**0.78**	9.09	7.52	2.13
	AnC (nmol m^−2^)	**0.86**	1.78	6.17	2.67	**0.85**	1.87	6.56	2.58
	Flv (nmol m^−2^)	**0.80**	11.75	15.60	2.24	**0.76**	12.60	16.85	2.04
	Phe (mL L^−1^)	**0.91**	5.57	3.15	3.33	**0.90**	5.94	3.38	3.16
1500-1150 cm^−1^	Chl*a* (mg m^−2^)	0.49	65.64	35.90	1.40	0.42	69.63	38.17	1.31
	Chl*b* (mg m^−2^)	0.53	30.97	14.45	1.46	0.49	32.99	15.47	1.40
	Chl*a*+*b* (mg m^−2^)	0.48	99.95	50.15	1.39	0.45	106.06	53.45	1.35
	Car (mg m^−2^)	0.54	21.04	10.95	1.47	0.51	22.30	11.64	1.43
	AnC (nmol m^−2^)	**0.91**	1.25	4.91	3.33	**0.89**	1.35	5.26	3.02
	Flv (nmol m^−2^)	**0.85**	8.38	13.10	2.58	**0.84**	8.98	13.98	2.50
	Phe (mL L^−1^)	**0.81**	11.58	4.37	2.29	**0.80**	12.20	4.64	2.24

**Table 3:** PLSR model in the predicted phase. Model goodness-of-fit (R^2^), offset, root mean square error (RMSEP), standard error of prediction (SEP), bias and ratio of performance to deviation (RPD) parameters from ATR-FTIR spectroscopy data of lettuce leaves. The bold represents statistically significant regression models (R^2^). The underline indicates a bad Bias calculated. Parameter abbreviations—see Table 1 or Abbreviation list.

Wavenumbers	Parameters	Predicted					
		R^2^	Offset	RMSEP	SEP	Bias	RPD
4000-400 cm^−1^	Chl*a* (mg m^−2^)	**0.71**	43.20	30.45	30.09	5.87	1.86
	Chl*b* (mg m^−2^)	**0.88**	11.64	9.30	9.27	−1.49	2.89
	Chl*a*+*b* (mg m^−2^)	**0.87**	39.58	29.80	29.25	−7.03	2.77
	Car (mg m^−2^)	**0.81**	6.10	5.69	5.60	−1.27	2.29
	AnC (nmol m^−2^)	**0.90**	0.73	5.27	5.33	**0.03**	3.16
	Flv (nmol m^−2^)	**0.87**	6.68	13.67	13.66	2.01	2.77
	Phe (mL L^−1^)	**0.93**	7.34	2.92	2.95	**0.04**	3.78
1500-1150 cm^−1^	Chl*a* (mg m^−2^)	0.61	54.29	26.65	26.53	−4.47	1.60
	Chl*b* (mg m^−2^)	0.58	30.98	70.99	31.36	−63.85	1.54
	Chl*a*+*b* (mg m^−2^)	0.61	88.39	38.23	38.35	−4.81	1.60
	Car (mg m^−2^)	0.66	19.17	7.92	7.84	−1.59	1.71
	AnC (nmol m^−2^)	**0.91**	1.43	4.98	5.02	**0.21**	3.33
	Flv (nmol m^−2^)	**0.89**	8.45	12.85	12.90	**1.27**	3.02
	Phe (mL L^−1^)	**0.79**	9.68	4.71	4.76	**0.03**	2.18

**Table 4:** The most responsive variable importance for projection (VIP) by wavenumbers selected between 4000 at 400 cm^−1^ and 1500 at 1150 cm^−1^ by ATR-FTIR spectroscopy according to the regression β-coefficient for quality parameter for the PLSR model to predict. Parameter abbreviations—see Abbreviation list or Table 1.

Parameters	4000-400 cm^−1^	1500-1150 cm^−1^	Number of Shared VIP	Most Responsive VIP by Wavenumbers (cm^−1^)	
				4000-400 cm^−1^	1500-1150 cm^−1^
Chl*a* (mg m^−2^)	13	10	3	2624; 2613; 2590; 2576; 2509; 2453; 2435; 2165; **1410**; **1392**; **1384**; 1369; 1361;	1486; 1477; 1461; 1450; **1415; 1396; 1382**; 1359; 1182; 1177;
Chl*b* (mg m^−2^)	16	12	3	2622; 2613; 2590; 2580; 2511; 2460; 2453; 2433; 2322; 2163; 2050; **1405**; 1400; **1384**; 1375; **1359**;	1498; 1486; 1479; 1463; 1452; 1415; 1402; **1384**; 1375; 1363; **1359**; 1182
Chl*a*+*b* (mg m^−2^)	14	11	4	2624; 2613; 2601; 2590; 2578; 2511; 2457; 2447; 2435; **1415**; 1404; **1388**; **1378**; **1359**;	1496; 1485; 1479; 1461; 1452; **1415**; 1398; **1386**; **1377**; **1361**; 1184;
Car (mg m^−2^)	13	11	4	2624; 2613; 2590; 2460; 2449; 2439; 2323; 2163; 2050; **1415**; **1384**; **1367**; **1359**;	1492; 1486; 1475; 1463; 1448; **1415**; 1392; **1386**; **1373**; **1361**; 1182;
AnC (nmol m^−2^)	15	12	1	2935; 2923; 2914; 2902; 2852; 1630; 1648; 1637; 1629; 1384; **1378**; 607; 599; 584; 578;	1485; 1477; 1463; 1459; 1452; 1415; 1407; 1392; **1377**; 1359; 1323; 1313;
Flv (nmol m^−2^)	13	13	0	2939; 2929; 2916; 2898; 2858; 2848; 1666; 1652; 1647; 1639; 1621; 1537; 1533;	1431; 1427; 1355; 1346; 1334; 1326; 1307; 1205; 1193; 1180; 1159; 1153; 1149;
Phe (mL L^−1^)	13	12	5	2929; 2921; 2914; **1404**; **1396**; 1384; **1375**; **1367**; 613; 599; 588; 572; 561;	1500; 1496; 1481; 1479; 1459; 1446; 1415; **1409**; **1402**; **1394**; **1373**; **1361**;

**Table 5:** Band assignments of ATR-FTIR spectra characteristic of the phenolic compounds and flavonoids in different spectra obtained by ATR-FTIR and the corresponding references.

Band (cm^−1^)	Origin	Assignment	Reference
3436	chlorophyll and carotenoids	*trans*–CH=CH– bond	[41,42]
2863	chlorophylls and carotenoids	–CH stretching	[11,14,43]
1735	lignin-phenolic compounds	stretching ν(C=O)	[44]
1650-1550	phenolic compounds and flavonoids	stretching ν(C=O)	[44,45]
1650-1400	flavonoids, phenolic compounds, and other phenolics	ring stretching –(C=O), ν(C=C) phenolic acids, ν(C–C) aromatic	[24,44]
1545	amide II	stretching (C=N) and (N–H)	[44]
1525	chlorophylls and carotenoids	(N–H bending)	[46]
1515	phenolic compounds and flavones phenyl ring	ring stretching C=C, aromatic conjugated C=C, N–H	[44,47]
1464-1429	presence of flavonoids and triterpenoids	–O–H	[47]
1455-1440	polysaccharides, lipids, proteins	asymmetric bending of –CH_2_ and –CH_3_	[44]
1440-1436	phenolic components	aromatic stretching ν(C–C) stretching ν(C=C)	[24,25]
1430-1420	polysaccharides, alcohol, carboxylic acid	bending –OH	[44]
1425-1380	aromatic compounds	stretching ν(C–C)	[7,14]
1300-1260	polyphenols and flavonoids	stretching ν(C–C)	[7,14]
1381	polysaccharides	symmetric bending –CH_2_ and –CH_3_	[48]
1293	wavenumber of the corresponds flavonoids	O–H	[47]
1260-1180	phenolic components	stretching –C–C–O	[48]
825-810	polyphenols	C–H out of plane bending vibrations of phenyl rings	[7]
790-760	phenolic components and flavonoids	aromatic ring vibration	[7,14,48]

## Data Availability

Not applicable.
